# Fatal congenital lobar emphysema in a puerpera: a case report and literature review

**DOI:** 10.1186/s12890-021-01787-x

**Published:** 2021-12-20

**Authors:** Fanyi Gan, Liang Xia, Yushang Yang, Qiang Pu, Lunxu Liu

**Affiliations:** 1 Department of Thoracic Surgery, West China Hospital, Sichuan University, No. 37 Guoxue Alley, Chengdu, 610041 Sichuan China; 2 Western China Collaborative Innovation Center for Early Diagnosis and Multidisciplinary Therapy of Lung Cancer, Sichuan University, Chengdu, 610041 China

**Keywords:** Congenital lobal emphysema, Adults, Computed tomography, Case report

## Abstract

**Background:**

Congenital lobal emphysema (CLE) is a developmental lung abnormality usually diagnosed in the neonatal period and is rarely observed in adults. Adults with CLE are usually asymptomatic and only a small fraction may present with coughing, recurrent pneumonia and respiratory distress. In imaging studies, the most frequently affected lobe of CLE is the left upper lobe, followed by the right middle lobe. However, multilobar involvement with severe mediastinal shift is extremely rare.

**Case presentation:**

We report a case of fatal CLE in a 28-year-old puerpera with postpartum respiratory failure. Chest computed tomography (CT) revealed emphysema of the right upper, middle and lower lobes resulting in adjacent atelectasis. Hyperinflation of the right upper lobe crossed the midline, leading to a deviation of the mediastinal structure to the left hemithorax and severe compression of the left lung.

**Conclusions:**

Early and timely diagnosis of CLE with routine follow-up is necessary for patients. CLE, especially with multilobar involvement or mediastinal shift, could be life-threatening and should be promptly and aggressively treated to prevent severe complications.

## Background

Congenital lobal emphysema (CLE) is a rare developmental lung abnormality usually diagnosed in the neonatal period, with an incidence of 1/20,000–30,000 [1]. Half of the CLE cases are idiopathic, and absence, hypoplasia or dysplasia of bronchial cartilage may occur in one-quarter of the patients [1]. Nearly all infants with CLE will develop symptoms and are diagnosed within the first 6 months of life [2, 3]; CLE in adults is quite rare. Adults with CLE are usually asymptomatic, and only a small fraction may present with coughing, recurrent pneumonia and respiratory distress [4, 5]. Imaging studies are regarded as the gold standard for the diagnosis of CLE, which is characterized by overinflation of the involved lobes [1]. The most frequently affected lobe is the left upper lobe, followed by the right middle lobe [6, 7]. However, multilobar involvement is extremely rare. To the best of our knowledge, CLE in adults with multilobar involvement has only been reported in a few cases [7, 8].

Here, we reported a typical and fatal CLE with all three lobes of the right lung involved and severe mediastinal shift in a puerpera. This case suggests us that CLE in adults can be life-threating; thus, early and timely diagnosis of CLE with routine follow-up is crucial.

## Case presentation

A 28-year-old puerpera suffering from severe dyspnoea was admitted to the emergency department. She had gave birth 3 days prior. A closed thoracic drainage procedure was performed for the diagnosis of right pneumothorax in a local hospital failed to relieve her symptom. She was transferred to our center due to declining respiratory status. The patient had a history of fatigue and exertional dyspnoea from her childhood, though she had never sought medical care previously. The dyspnoea suddenly worsened in the third trimester. She was a nonsmoker and free from other medical history.

This patient was lethargic on admission, and auscultation of the chest showed an absence of breath sounds in the right lung and decreased breath sounds in the left lung. Arterial blood gas revealed a PO_2_ of 106.3 mmHg and a PCO_2_ of 113.1 mmHg upon 53% oxygen concentration inhalation. Emergency tracheal intubation was performed considering the diagnosis of respiratory failure and her state-of-consciousness. She was then transferred to the intensive care unit.

**Fig. 1:**
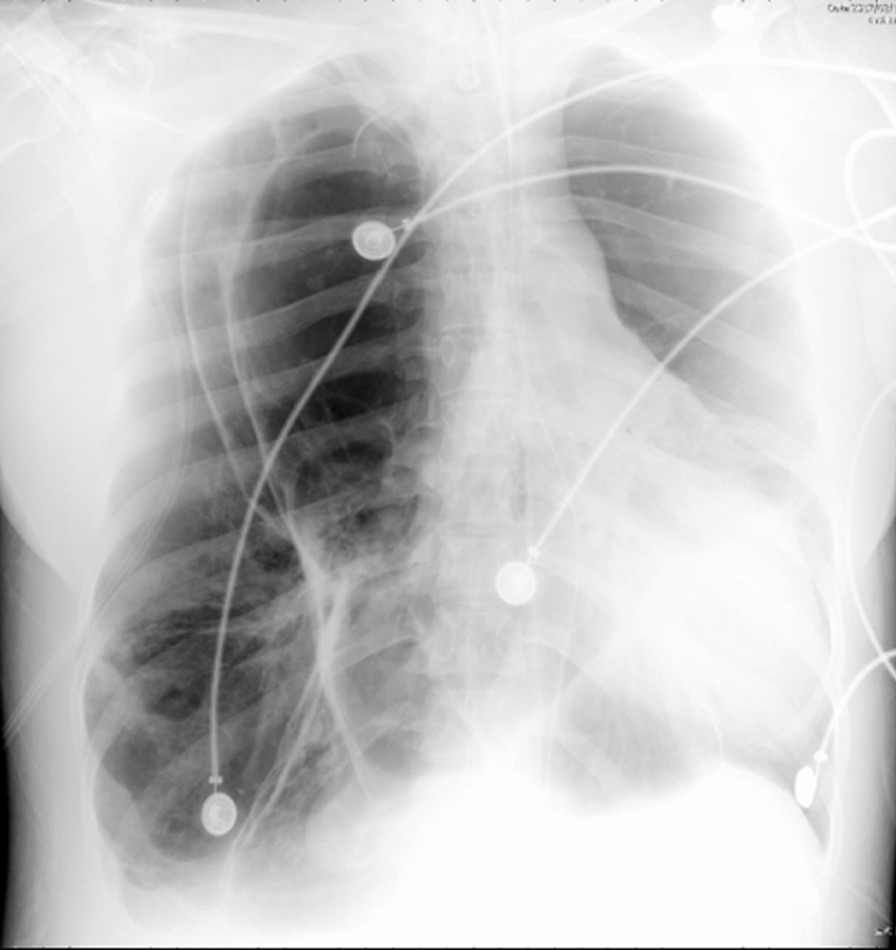
Chest X-ray revealing increased radiolucency of almost the right lung, and the mediastinum shifted to the left

**Fig. 2:**
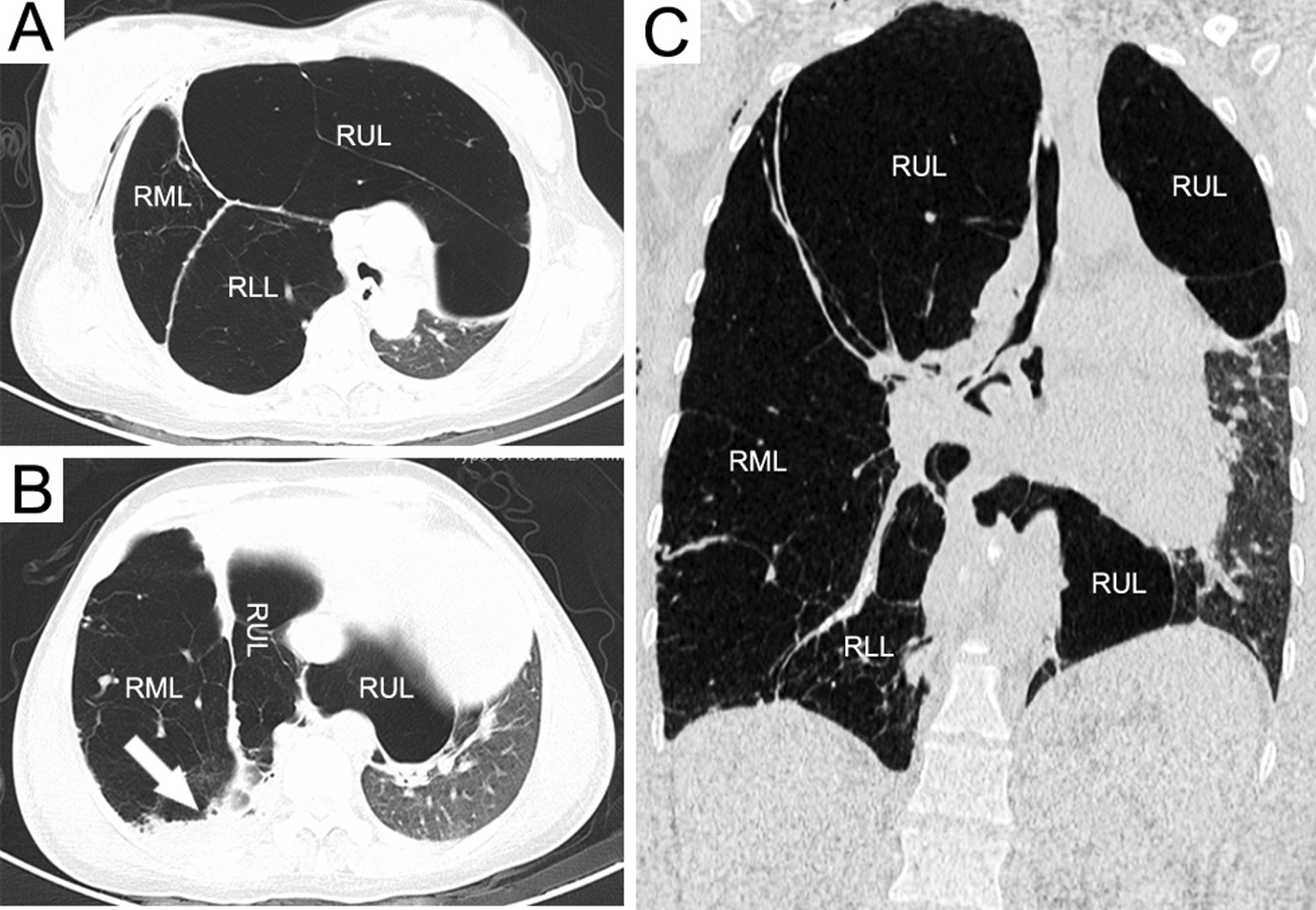
Chest computed tomography (CT) displaying emphysema of the right upper, middle and lower lobes resulting in adjacent atelectasis (arrow); hyperinflation of the right upper lobe crossed the midline, leading to a deviation of the mediastinal structure to the left hemithorax and severe compression of the left lung. A and B, Axial chest CT scans; C, Coronal chest CT scan. RUL, right upper lobe; RML, right middle lobe; RLL, right lower lobe

Chest radiograph revealed increased radiolucency of almost the whole right lung, and the mediastinum shifted to the left (Fig. 1). Chest computed tomography (CT) displayed emphysema of the right upper, middle and lower lobes resulting in adjacent atelectasis. Hyperinflation of the right upper lobe crossed the midline, leading to a deviation of the mediastinal structure to the left hemithorax and severe compression of the left lung (Fig. 2). Flexible bronchoscopy revealed no obstructive intrabronchial lesions. The clinical diagnosis of congenital lobar emphysema (CLE) was established in combination with the clinical history and imaging examinations.

Since the patient could not tolerate one-lung ventilation, we planned to perform pulmonary resection assisted by extracorporeal membrane oxygenation. However, her legal representative refused the recommendation, and unfortunately, she eventually succumbed to respiratory failure and pulmonary infection after failed conservative management.

## Discussion and conclusions

CLE is a developmental abnormality usually diagnosed in the neonatal period and is rarely diagnosed in adulthood. Few cases of adults with CLE have been reported before, and we have summarized the characteristics of them in Table 1 [5, 7–11]. The gold standard for the diagnosis of CLE relies on imaging examinations [1]. CLE is characterized by overinflation of the involved lobes, with or without attenuated and displaced pulmonary vessels on imaging studies [4, 6, 8]. Multilobar involvement in CLE is extremely rare. Shen et al. reported CLE in an elderly patient with involvement of the right upper and middle lobes [8]. Our case presented a typical and fatal CLE with all three lobes of the right lung involved and severe mediastinal shift in a puerpera.

**Table 1:** Characteristics of reported congenital lobar emphysema in adults

Literature	Patient	The way of diagnosis	Involved lobes	Mediastinal shift
Sadaqat et al. [9]	A 19-year-old man	Chest radiograph and Chest CT	Right upper lobe	Yes
Ogul et al. [10]	A 22-year-old man	Chest CT	Right middle lobe	Yes
Muramatsu et al. [11]	A 31-year-old woman	Chest radiograph and Chest CT	Right middle lobe	No
Sasieta et al. [7]	A 37-year-old man	Chest radiograph and Chest CT	Right middle lobe	Yes
King et al. [5]	A 28-year-old woman	Chest radiograph and Chest CT	Left lower lobe	No
Shen et al. [8]	A 78-year-old man	Chest radiograph and Chest CT	Right upper and middle lobes	No
Our case	A 28-year-old puerpera	Chest radiograph and Chest CT	Right upper, middle and lower lobes	Yes

The treatment choice for adults is dependent on the clinical severity, mainly including either conservative management for patients with mild symptoms or lobectomy for patients with disease progression [1].

Pregnancy-specific conditions like amniotic fluid embolism, preeclampsia can lead to respiratory failure in the peripartum period. Besides, pulmonary conditions including asthma, infection, thromboembolism and CLE are also causes of peripartum respiratory failure [12]. This patient developed respiratory failure after giving birth, indicating that severe CLE can be life-threatening in the peripartum period. Therefore, early and timely diagnosis of CLE is extremely important, and adult patients diagnosed with CLE should be followed routinely even when they are in stable condition. Pulmonary resection is an alternative treatment modality [1, 7]. For this patient, we considered surgical intervention to correct the mediastinal shift and assist in re-expansion of the compressed lung, since a previous report indicated positive re-expansion of a long-term compressed lobe after surgery in a patient with CLE [7].

Finally, the lessons we can learn from this case include that early and timely diagnosis of CLE with routine follow-up is necessary. CLE, especially with multilobar involvement or mediastinal shift, can be life-threatening and should be promptly and aggressively treated to prevent severe complications.

## Data Availability

All data and materials are provided in the manuscript.

## Abbreviations

CLE: Congenital lobar emphysema
RUL: Right upper lobe
RML: Right middle lobe
RLL: Right lower lobe

## References

[1] Demir OF, Hangul M, Kose M (2019). Congenital lobar emphysema: diagnosis and treatment options. Int J Chron Obstruct Pulmon Dis.

[2] Karnak I, Senocak ME, Ciftci AO, Büyükpamukçu N (1999). Congenital lobar emphysema: diagnostic and therapeutic considerations. J Pediatr Surg.

[3] Thakral CL, Maji DC, Sajwani MJ (2001). Congenital lobar emphysema: experience with 21 cases. Pediatr Surg Int.

[4] Kumar B, Agrawal LD, Sharma SB (2008). Congenital bronchopulmonary malformations: a single-center experience and a review of literature. Ann Thorac Med.

[5] King N, Ramesh SS, Essandoh M, Merritt RE (2017). Near complete obliteration of the left hemithorax by congenital lobar emphysema in an adult. Ann Thorac Surg.

[6] Abushahin AM, Tuffaha AS, Khalil NK, Ismeal AM (2012). Bilateral congenital lobar emphysema: a rare cause for respiratory distress in infancy. Ann Thorac Med.

[7] Sasieta HC, Nichols FC, Kuzo RS, Boland JM, Utz JP (2016). Congenital lobar emphysema in an adult. Am J Respir Crit Care Med.

[8] Shen C, Che G (2020). Congenital lobar emphysema in an elderly patient. Am J Respir Crit Care Med.

[9] Sadaqat M, Malik J, Karim R (2011). Congenital lobar emphysema in an adult. Lung India Off Organ Indian Chest Soc.

[10] Ogul H, Sevketbeyoglu H, Ozgokce M, Alper F (2012). Congenital lobar emphysema association with double superior vena cava and horseshoe kidney. Ann Thorac Surg.

[11] Muramatsu T, Furuichi M, Nishii T, Ishimoto S, Shiono M (2013). Lobar emphysema with pneumothorax in an adult: report of a case. Surg Today.

[12] Lapinsky S (2015). Acute respiratory failure in pregnancy. Obstet Med.

